# Supplementary material for: Perspectives of Positively Charged Nanocrystals of Tedizolid Phosphate as a Topical Ocular Application in Rabbits
Source: Molecules. 2022 Jul 20;27(14):4619. doi: 10.3390/molecules27144619 (PMC9325164; doi:10.3390/molecules27144619)
Supplement: Supplementary file 1 [file molecules-27-04619-s001.zip › molecules-1792011-supplementary.pdf]

Supplementary Data

Perspectives of Positively Charged Nanocrystals of Tedizolid Phosphate as Topical Ocular Application in Rabbits

Abdullah Alshememry [1#], Musaed Alkholief [1#], Mohd Abul Kalam [1], Mohammad Raish [1], Raisuddin Ali [1], Sulaiman S. Alhudaithi [1], Muzaffar Iqbal [2] and Aws Alshamsan [1,*]

[1] Department of Pharmaceutics, College of Pharmacy, King Saud University, Riyadh 11451, Saudi Arabia; makalam@ksu.edu.sa (M.A.K); mraish@ksu.edu.sa (M.R); ramohammad@ksu.edu.sa (R.A) aalshememry@ksu.edu.sa (A.A); malkholief@ksu.edu.sa (M.A)

[2] Department of Pharmaceutical Chemistry, College of Pharmacy, King Saud University, Riyadh 11451, Saudi Arabia; muziqbal@ksu.edu.sa (M.I)

***** Correspondence: aalshamsan@ksu.edu.sa

[#] These authors contributed equally to this work.

Supplementary Tables

Table S1: Grading system for ocular irritation test

Cornea		
**Lesion**		**Score**
**a. Opacity-Degree of density (area which is most dense is taken for reading)**		
No ulceration or opacity		**0**
Scattered or diffuse area – details of iris clearly visible		**1**
Easily discernible translucent areas, details of iris slightly obscured		**2**
Opalescent areas, no details of iris visible, size of pupil barely discernible		**3**
Opaque, iris invisible		**4**
**b. Area of cornea involved**		
One quarter (or less) but not zero		**1**
Greater than one quarter but less than one half		**2**
Greater than one half but less than three quarters		**3**
Greater than three quarters up to whole area		**4**
**Score equals (a x b x 5):**	**Total maximum =**	**80**
**Iris**		
**Lesion**		**Score**
**a. Values**		
Normal		**0**
Folds above normal, congestion, swelling, circumcorneal injection (any one or all of these or combination of any thereof), iris still reacting to light (sluggish reaction is positive)		**1**
No reaction to light, hemorrhage; gross destruction (any one/ all of these)		**2**
**Score equals (a x 5):**	**Total maximum =**	**10**
**Conjunctiva**		
**Lesion**		**Score**
**a. Redness (refers to palpebral conjunctiva only)**		
Vessels normal		**0**
Vessels definitely injected above normal		**1**
More diffuse, deeper crimson red, individual vessels not easily discernible		**2**
Diffuse beefy red		**3**
**b. Chemosis**		
No swelling		**0**
Any swelling above normal (includes nictitating membrane)		**1**
Obvious swelling with partial eversion of the lids		**2**
Swelling with lids about half closed		**3**
Swelling with lids about half closed to completely closed		**4**
**c. Discharge**		
No any discharge		**0**
Any amount different from normal (does not include small amount observed in inner canthus of normal animals)		**1**
Discharge with moistening of the lids and hairs just adjacent to the lids		**2**
Discharge with moistening of the lids and considerable area around the eye		**3**
**Score equals (a + b + c) x 2:**	**Total maximum =**	**20**

Table S2: Classification of eye irritation scoring system

Classification of Irritation	Maximum Mean Total Score (MMTS*)
None	0.0-0.5
Practically none	0.6-2.5
Minimally	2.6-15.0
Mildly	15.1-25.0
Moderately	25.1-50.0
Severely	50.1-80.0
Extremely	80.1-100.0
Maximally	100.1-110.0

Figure S1

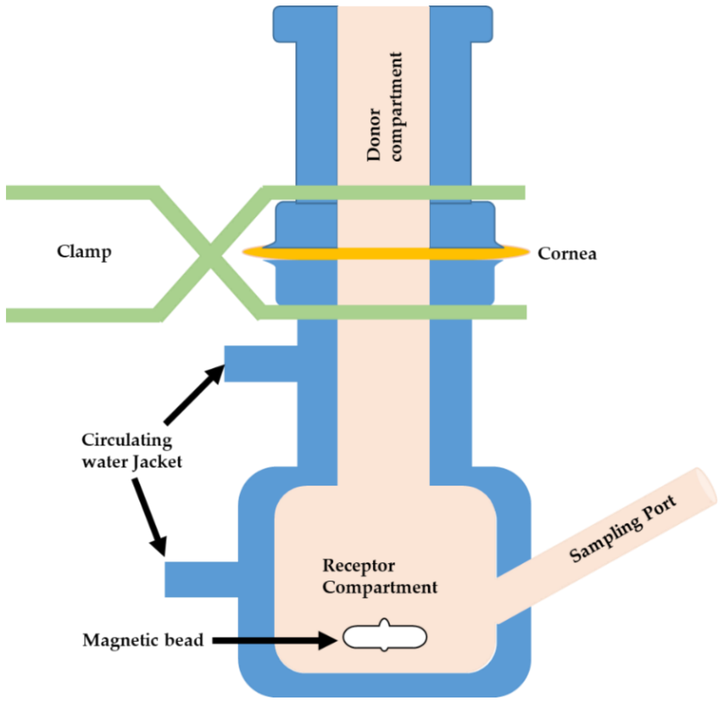

Figure S1: Schematic representation of Franz Diffusion cell
